# Intratumoral heterogeneity analysis reveals hidden associations between protein expression losses and patient survival in clear cell renal cell carcinoma

**DOI:** 10.18632/oncotarget.16965

**Published:** 2017-04-08

**Authors:** Wei Jiang, Essel Dulaimi, Karthik Devarajan, Theodore Parsons, Qiong Wang, Raymond O'Neill, Charalambos Solomides, Stephen C. Peiper, Joseph R. Testa, Robert Uzzo, Haifeng Yang

**Affiliations:** ^1^ Department of Pathology, Anatomy and Cell Biology, Thomas Jefferson University, Philadelphia, PA, United States; ^2^ Department of Pathology, Fox Chase Cancer Center, Philadelphia, PA, United States; ^3^ Biostatistics, Fox Chase Cancer Center, Philadelphia, PA, United States; ^4^ Cancer Biology, Fox Chase Cancer Center, Philadelphia, PA, United States; ^5^ Department of Surgical Oncology, Fox Chase Cancer Center, Philadelphia, PA, United States

**Keywords:** clear cell renal cell carcinoma, immunohistochemistry, intratumoral heterogeneity, overall survival, SWI/SNF

## Abstract

Intratumoral heterogeneity (ITH) is a prominent feature of kidney cancer. It is not known whether it has utility in finding associations between protein expression and clinical parameters. We used ITH that is detected by immunohistochemistry (IHC) to aid the association analysis between the loss of SWI/SNF components and clinical parameters.160 ccRCC tumors (40 per tumor stage) were used to generate tissue microarray (TMA). Four foci from different regions of each tumor were selected. IHC was performed against PBRM1, ARID1A, SETD2, SMARCA4, and SMARCA2. Statistical analyses were performed to correlate biomarker losses with patho-clinical parameters. Categorical variables were compared between groups using Fisher's exact tests. Univariate and multivariable analyses were used to correlate biomarker changes and patient survivals. Multivariable analyses were performed by constructing decision trees using the classification and regression trees (CART) methodology. IHC detected widespread ITH in ccRCC tumors. The statistical analysis of the “Truncal loss” (root loss) found additional correlations between biomarker losses and tumor stages than the traditional “Loss in tumor (total)”. Losses of SMARCA4 or SMARCA2 significantly improved prognosis for overall survival (OS). Losses of PBRM1, ARID1A or SETD2 had the opposite effect. Thus “Truncal Loss” analysis revealed hidden links between protein losses and patient survival in ccRCC.

## INTRODUCTION

### What is intratumoral heterogeneity (ITH)?

One or a few cancerous cells with a few founding mutation(s) are the origins of tumors, then during tumor development additional mutations occurred to aid progression [1]. Consequently in many cancers different regions of a tumor share the same founding mutations but have different mutations that happened later. This regionally mixed mutational landscape is defined as Intratumoral Heterogeneity (ITH). ITH was discovered in many types of cancers including leukemia [2], glioblastoma [3], colon [4], pancreatic [5], ovarian [6], breast [7] and clear cell renal cell carcinoma (ccRCC) cancers [8, 9]. ITH suggest that tumor development occurs in a branched fashion instead of a linear one.

### ITH and mutations in ccRCC

In ccRCC the loss of function of von-Hippel Lindau tumor suppressor (*VHL*) happens in around 80% of tumors. It is inactivated through DNA mutations or promoter hypermethylation, and it is the founding mutation for ccRCC [10]. The familial VHL syndrome, which includes ccRCC as one of the lesions, is caused by germline *VHL* mutations. In recent years, large-scale sequencing studies identified additional mutated tumor suppressors [11–13]. Around 40% of ccRCC tumors were found to harbor mutations in polybromo-1 (*PBRM1*), a component of a SWI/SNF chromatin-remodeling complex [11]. In addition, 10–15% of ccRCC tumors have inactivating mutations in either BRCA1-associated protein 1 (*BAP1*) or SET domain containing 2 (*SETD2*), a histone deubiquitinase and a histone methyltransferase respectively [12].

Gerlinger et al discovered that ITH was very prevalent in ccRCC [9]. They also identified convergent phenotypic evolution. In the same tumor, distinct mutations at different parts of the tumor could inactivate the same tumor suppressor genes such as *SETD2*, Phosphatase And Tensin Homolog (*PTEN*), and Lysine Demthylase 5C (*KDM5C/JARID1C*). In their analysis of eight kidney cancer samples, only chromosome 3p loss and *VHL* aberrations were present in all the cases. They were called truncal losses (root and ubiquitous losses) [8]. In tumors with *PBRM1* mutations, half of them were truncal [8].

### Can ITH be examined by IHC? can ITH be useful in predicting clinical outcome?

The ITH in ccRCC was primarily studied with Next Gen Sequencing (NGS). It provided high quality data and great resolution, but it is expensive and labor intensive. Consequently the number of the analyzed samples is small which prevented statistical analysis to correlate with clinical parameters. We investigated whether IHC could successfully characterize ITH. We further investigated whether the ITH analysis at a much larger scale could reveal hidden correlations between the loss of biomarkers and clinical parameters.

## RESULTS

### Immunohistochemical analysis of ccRCC foci on tissue microarray (TMA)

**Figure 1:**
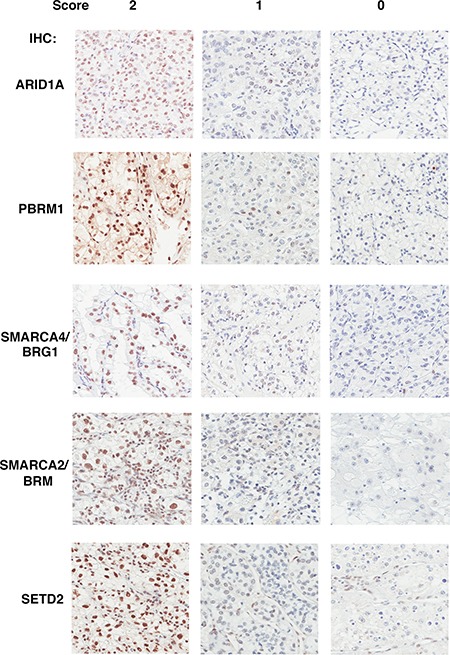
The Immunohistochemical analysis of ARID1A, PBRM1, SMARCA4, SMARCA2, SETD2 in ccRCC foci Representative foci stained for different markers showing scores of 2, 1, 0 (left, middle, and right). The staining of stromal and immunological cells serves as internal positive controls.

The demographic, pathological and clinical parameters of the ccRCC patients we selected for this study are presented in Table 1. We excised four foci from different areas from each tumor to construct TMA. In our previous publication we examined the specificity of the antibodies with cells expressing shRNA against target proteins and found them to be specific [14]. In addition many of these antibodies revealed expression losses when mutations in the target genes were detected [15–19]. With validated antibodies we stained five sets of the TMA. We found that all five proteins were stained primarily in the nucleus (Figure 1). This is consistent with the known roles of these proteins as chromatin regulators.

**Table 1:** Characteristics of ccRCC patients included in this study

Number of patients	160
**Age [average (range)]**	59.7 (23–82)
**Gender**	
Male	118
Female	42
**Race**	
African American	9
Caucasian	148
Other	3
**Grade**	
Grade 1	11
Grade 2	37
Grade 3	71
Grade 4	40
**Path T Stage**	
T1	47
T2	51
T3	60
T4	2
**N Stage**	
N0	118
N1 and N2	13
NX	29
**M Stage**	
M0	102
M1	37
MX	21
**TNM Stage**	
1	40
2	40
3	40
4	40
**Histology**	
Clear Cell only	160

### The summary of protein expression losses in tumors and foci

To be consistent with rule on DNA mutation calling in tumors, we decided that if the expression of a marker was gone in one focus out of four foci from a tumor, then that tumor had a loss of expression of that marker. The detailed tally of the protein expression loss was described before [14]. We found that 31% of tumors lost expression of PBRM1. In addition, 51% of them lost ARID1A, 14% of them lost SETD2, 15% of them lost SMARCA4, and 38% of them lost SMARCA2 expressions (Table 2). If the loss of protein expression was calculated with foci, 17%, 32%, 6.1%, 6.9% and 22% of foci lost the expressions of PBRM1, ARID1A, SETD2, SMARCA4, and SMARCA2 respectively (Table 2).

**Table 2:** Summary of protein expression losses in ccRCC tumors

	Tumors with protein expression loss	Foci with protein expression loss
PBRM1	31% (49/160)	17% (108/638)
ARID1A	51% (81/160)	32% (202/638)
SETD2	14% (23/160)	6.1% (39/638)
SMARCA4	15% (24/160)	6.9% (44/638)
SMARCA2	38% (61/160)	22% (143/638)

### The truncal loss analysis revealed hidden links between biomarker losses and tumor stages

**Figure 2:**
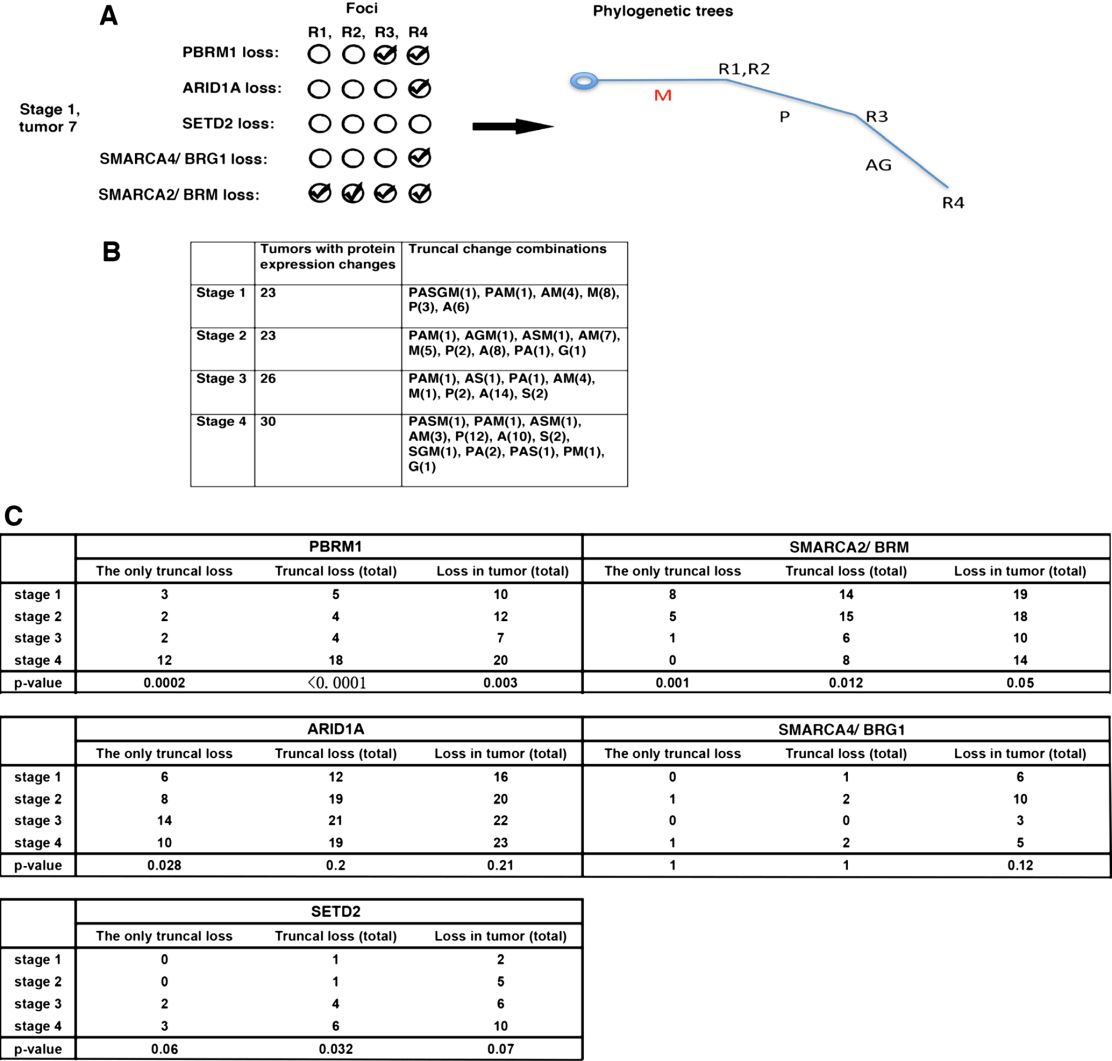
The truncal loss analysis revealed hidden links between protein loss and tumor stages (**A**) How a phylogenetic tree was constructed. A: ARID1A loss; M: SMARCA2 loss; P: PBRM1 loss, G: SMARCA4 loss; S: SETD2 loss. (**B**) Truncal losses of the markers at each stage, either alone or in combination, were presented. (**C**) Fisher's exact tests were performed to calculate the *p* values of the associations between the protein marker losses and stages.

The relationships between different molecular events can be inferred by clonal ordering [20], and a phylogenetic tree can be constructed to represent this. If a molecular event is a founding one, it will be present in most regions of a tumor. We call it a truncal (early or root) change. Conversely, if a molecular event arises late during tumor development, this change might be only detected in one or two foci. We call it a branch (late) change. Tumor #7 from the stage 1 group provided an example: the SMARCA2 loss was a truncal event, PBRM1 loss was a branch event, while ARID1A and SMARCA4 losses were branch events that happened even later (Figure 2A).

Next we examined the truncal changes that occurred in these tumors. Each tumor stage was represented by 40 tumors, and 23, 23, 26, 30 cases from stage 1 to 4 had protein expression losses respectively (Figure 2B). For brevity, we called the protein losses A (ARID1A loss), P (PBRM1 loss), S (SETD2 loss), G (SMARCA4/BRG1 loss), M (SMARCA2/BRM loss). We grouped the protein losses into three camps: Only Truncal Loss (it includes tumors with truncal loss that is the only truncal loss), Truncal Loss (Total) (it includes tumors with truncal loss, either alone or in combination), or the Loss in Tumor (Total) (it includes all the tumors with protein losses). We then used Fisher's exact tests to examine whether the biomarker losses were statistically associated with high tumor stage (stage 4). In the case of PBRM1, the loss frequencies increased with stage and the associations between truncal loss groups with high stage had much smaller *p* values than that of Loss in Tumor (Total), which suggested higher confidence (Figure 2C). For SMARCA2, the loss frequency decreased when stage increased. The truncal loss groups had very small *p* values, while that of Loss in Tumor (Total) was a borderline 0.05 (Figure 2C). For ARID1A, the higher stages had more protein losses, but just the Only Truncal Loss group had a statistically significant association with high stage (Figure 2C). SMARCA4 loss did not show any statistically significant association with high stage (Figure 2C). As for SETD2, only the Truncal Loss (Total) group was significantly associated with high stage (*p* = 0.032) (Figure 2C).

### The truncal loss analysis reveals hidden associations between protein losses and patient survival

**Figure 3:**
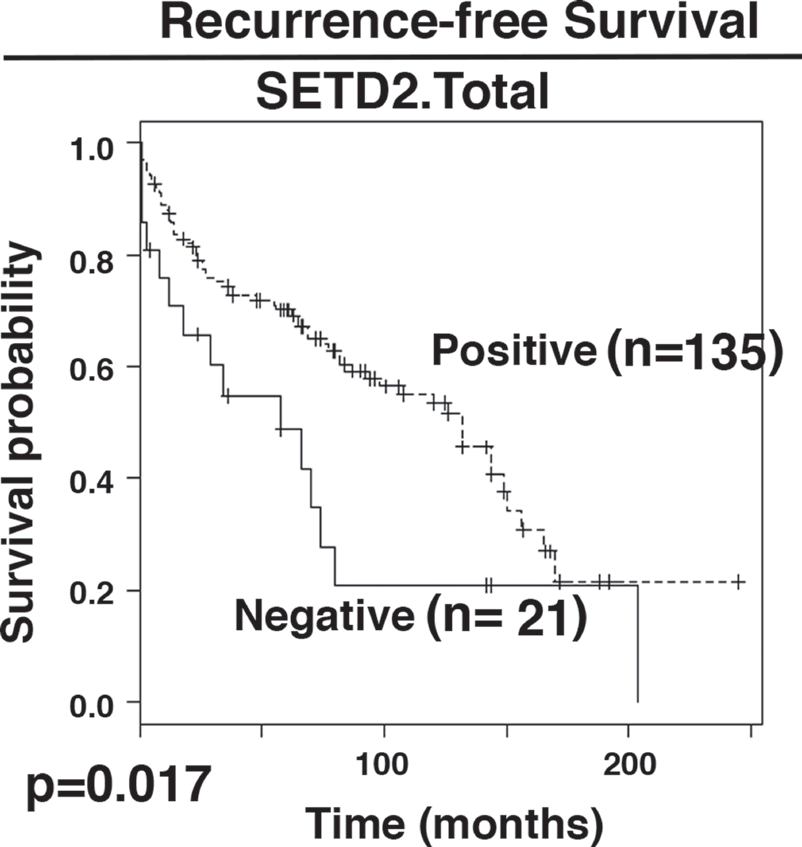
Kaplan-Meier analysis of recurrent free survival The survival curves were calculated based on SETD2 staining: positive (1) and negative (0). Associated log-rank *p* value was indicated. n: number of cases.

It is not known whether truncal losses of protein markers would reveal statistically different associations with patient survival than those of total losses. Cox proportional hazards (PH) models were utilized to correlate recurrence-free survival (RFS) with biomarker losses. For the protein losses, two groups of protein losses were used for analysis: one included all the truncal losses (Proteinname.Truncal). The other one included all the protein losses (Proteinname.Total). In univariate analyses, SETD2.Total, SMARCA2.Total, SMARCA4.Truncal, and SMARCA2.Truncal displayed a significant association with RFS with *p* values near or below 0.05 while SMARCA4.Total and SETD2.Truncal showed marginally significant associations (Table 3). In further multivariable analysis, only SETD2.Total's association with RFS remained statistically significant. The Kaplan-Meier curve showed that patients that lost SETD2 staining in tumors had shorter RFS (Figure 3). Thus, most of the markers do not appear to be associated with RFS and the ITH analysis did not help.

**Table 3:** Univariate and multivariable analyses of indicated biomarker losses and their associations with recurrence-free survival

Recurrence-free survival		
Univariate analysis	All patients (160)	
	Hazard Ratio (95% CI)	*p* value
ARID1A.Total	0.93 (0.59–1.46)	0.754
SETD2.Total	0.50 (0.28–0.89)	0.017
SMARCA4.Total	1.98 (0.98–4.01)	0.056
SMARCA2.Total	1.83 (1.12–3.0)	0.015
PBRM1.Truncal	0.79 (0.55–1.14)	0.210
SETD2.Truncal	0.72 (0.51–1.02)	0.065
SMARCA4.Truncal	1.85 (1.06–3.23)	0.031
SMARCA2.Truncal	1.79 (1.31–2.46)	0.0002
**Multivariable analysis**	**All patients (160)**	
	**Hazard Ratio (95% CI)**	***p* value**
SETD2.Total	0.27 (0.08–0.90)	0.034
SMARCA4.Total	1.63 (0.55–4.82)	0.380
SMARCA2.Total	1.57 (0.74–3.36)	0.244
SETD2.Truncal	1.13 (0.61–2.11)	0.695
SMARCA4.Truncal	1.35 (0.64–2.85)	0.429
SMARCA2.Truncal	1.32 (0.85–2.07)	0.218

The biomarker loss was subjected to multivariable analysis if its *p* value was below 0.1. CI = confidence interval.

**Figure 4:**
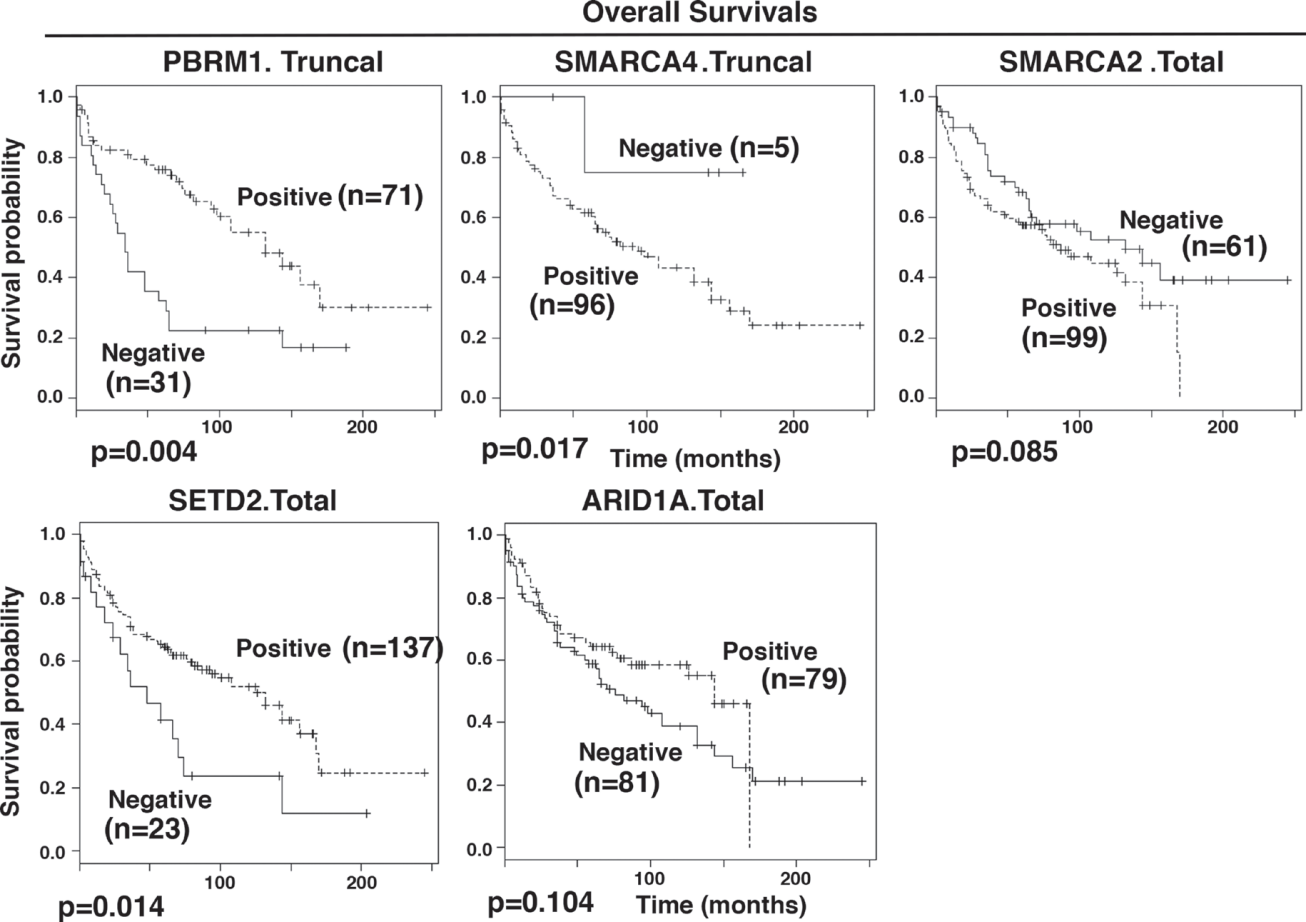
Kaplan-Meier analysis of overall survival The survival curves were calculated based on biomarker staining: positive (1) and negative (0). Associated log-rank *p* values were indicated. n: number of cases analyzed.

The overall survival (OS) is clinically crucial and has greater importance than RFS. In univariate analyses, tests of association between ARID1A.Total, SETD2.Total, SMARCA2.Total, PBRM1.Truncal, SMARCA4.Truncal, SMARCA2.Truncal and OS showed *p* values below or near 0.1 (Table 4). They were used for further multivariable analysis. With the exception of SMARCA2. Truncal, all the other biomarker losses showed a statistically significant association with OS. It was found that losses of SMARCA4.Truncal or SMARCA2.Total were associated with significantly better prognosis for patients (hazard ratio of 2.55 and 3.59 respectively), while losses of ARID1A.Total, PBRM1.Truncal, or SETD2.Total were associated with worse prognosis (hazard ratio of 0.23, 0.42, and 0.3 respectively). Truncal loss counts only the cases with truncal protein expression losses, while the Total loss includes all the tumors with protein expression losses. The Kaplan-Meier curves showed the same trends (Figure 4).

**Table 4:** Univariate and multivariable analyses of indicated biomarker losses and their associations with overall survival

Overall survival		
Univariate analysis	All patients (160)	
	Hazard Ratio (95% CI)	*p* value
ARID1A.Total	0.69 (0.44–1.08)	0.104
SETD2.Total	0.50 (0.29–0.87)	0.014
SMARCA4.Total	1.55 (0.82–2.94)	0.182
SMARCA2.Total	1.50 (0.94–2.38)	0.085
PBRM1.Truncal	0.60 (0.42–0.85)	0.004
SETD2.Truncal	0.77 (0.54–1.09)	0.139
SMARCA4.Truncal	1.97 (1.13–3.45)	0.017
SMARCA2.Truncal	1.60 (1.18–2.17)	0.002
**Multivariable analysis**	**All patients (160)**	
	**Hazard Ratio (95% CI)**	***p* value**
ARID1A.Total	0.23 (0.09–0.55)	0.001
SETD2.Total	0.30 (0.15–0.58)	0.0003
SMARCA2.Total	3.59 (1.62–7.94)	0.002
PBRM1.Truncal	0.42 (0.29–0.63)	2.45e-05
SMARCA4.Truncal	2.55 (1.38–4.71)	0.003
SMARCA2.Truncal	0.99 (0.66–1.48)	0.948

The biomarker loss was subjected to multivariable analysis if its *p* value was below 0.1. CI = confidence interval.

**Figure 5:**
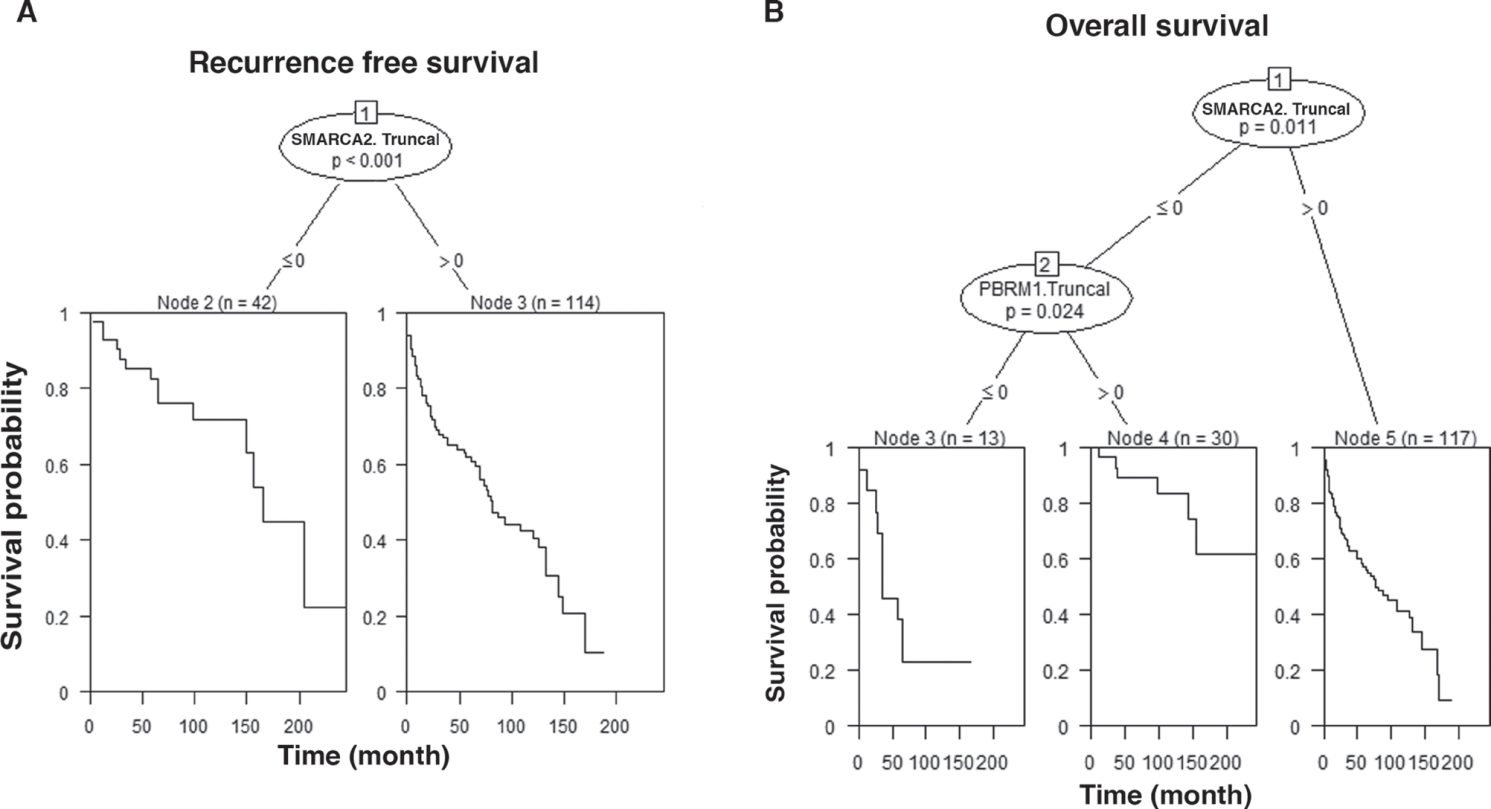
CART The decision trees and the survival curves are calculated based on staining of indicated biomarkers: positive (> 0) and negative (≤ 0). Associated *p* value was indicated. n: number of cases analyzed. Truncal changes were derived from ITH analysis. (**A**) CART analysis of biomarkers with RFS; (**B**) CART analysis of biomarkers with OS.

In order to further analyze our data, classification and regression trees (CART) methodology was applied to construct decision trees with multivariable analyses. RFS analysis revealed that patients whose SMARCA2. Truncal staining was negative (≤ 0) had significantly longer recurrence free survival than patients with positive SMARCA2 staining (Figure 5A). OS analysis revealed that patients whose SMARCA2.Truncal and PBRM1.Truncal staining were both negative showed the worst overall survival while those with negative SMARCA2.Truncal staining and positive PBRM1. Truncal staining showed the best overall survival (Figure 5B).

## DISCUSSION

**Figure 6:**
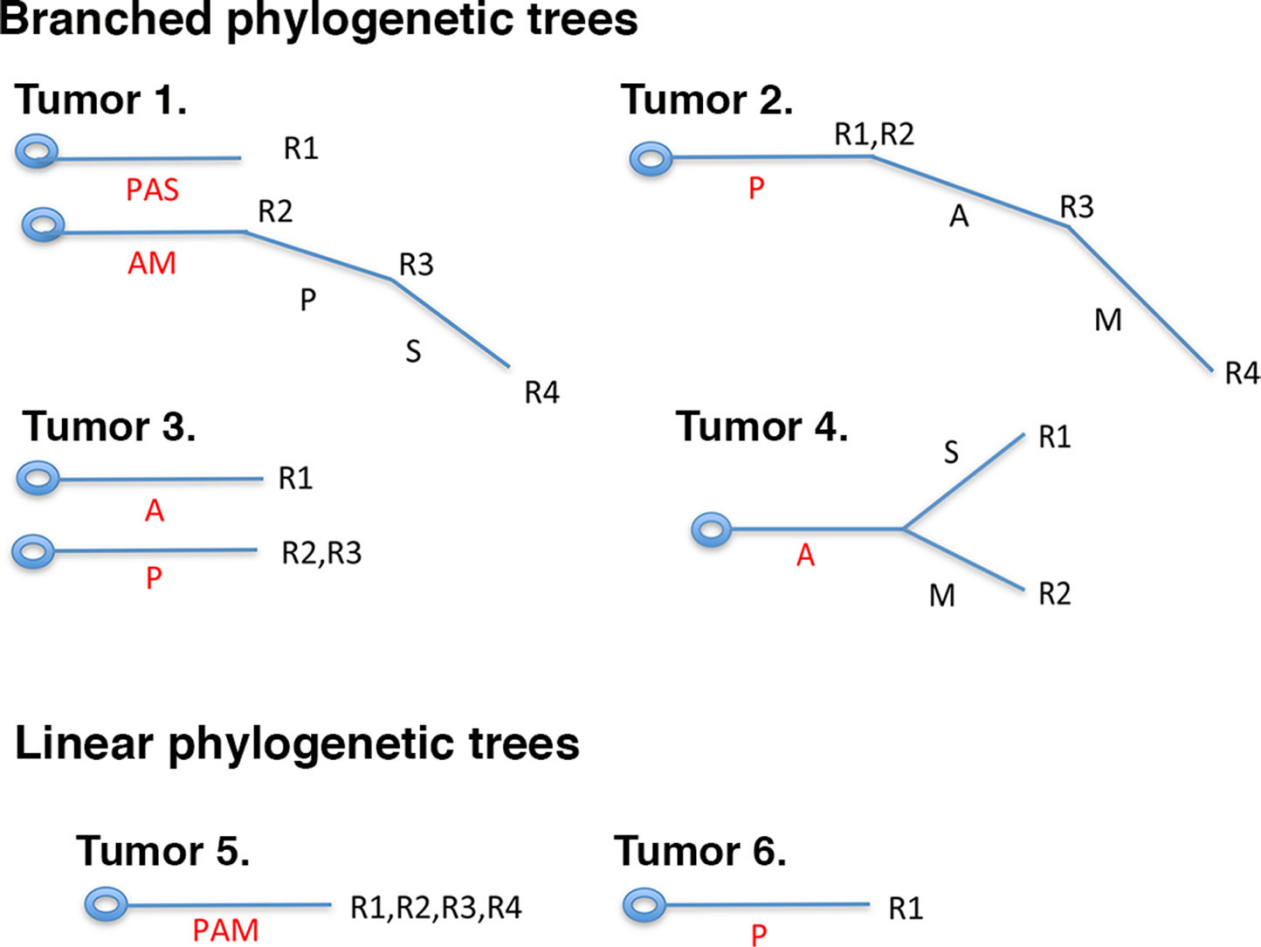
Branched or linear fashions of protein losses in ccRCC tumors The branched fashion of protein losses can have two roots (tumors 1, 3) or one root (tumors 2, 4), but at different parts of the same tumor the protein losses were not uniform, and some protein losses were only present in a subset of tumor foci. In the case of linear fashion of protein loss, one or multiple proteins were lost at once in one or multiple foci of the tumors (tumors 5, 6).

The loss of protein expression of important cancer genes can occur in a branched fashion or linear fashion in different parts of the same tumor (Figure 6). The branched fashion occurs in the vast majority of ccRCC tumors, constituting the intratumoral heterogeneity phenomena. Intratumoral heterogeneity is one of the major reasons that cancers are hard to eradicate. A major trend in cancer therapies, precision medicine, based upon the notion that the tumors in each person need a few major driving DNA mutations for tumorigenesis and tumor maintenance, and the drugs that hit the vulnerabilities conferred by such mutations will lead to clinical efficacy. This was proven true in many cases: Gleevec for chronic myeloid leukemia (CML) [21] and gastrointestinal stromal tumors (GIST) [22], Gefitinib for non-small cell lung cancer carrying hyperactive and mutated EGFR [23], and Vemurafenib for melanoma [24]. These drugs do not kill dividing cells non-discriminately so they tend to be quite effective with mild side effects. Unfortunately, in most cases tumors would develop drug resistance sooner or later. In a certain tumor, ITH could mean that a small percentage of the cancer cells do not carry the driving mutations, so over time they would grow up after treatment. Alternatively, some cancer cells might also harbor other mutations or epigenetic changes that render them drug resistant [25].

The cost of DNA sequencing prevented it from being applied to large-scale analysis of ITH. We show that IHC analysis can describe ITH at a large scale [14]. With the result we first examined whether ITH is useful in finding correlations between protein losses and high tumor stage. For ARID1A and SETD2, the statistically significant associations between marker losses and tumor stages would have been missed if truncal loss analysis were not performed (Figure 2). Thus the ITH analysis is useful here. However, it is highly likely that many tumor-derived mutations in the cancer genes, especially the point mutations, do not lead to protein expression loss, so mutational analysis will improve the sensitivity of analysis.

Next we examined the links between marker losses and recurrence-free survival (RFS). Only SETD2.Total showed a statistical meaningful association with RFS, and the loss of SETD2 led to shorter RFS (Table 3 and Figure 3). Thus these markers are mostly not very useful in predicting RFS, and ITH analysis did not help either.

The overall survival (OS) measures how long the patients survive after diagnosis. After multivariable analysis, ARID1A.Total, SETD2.Total, SMARCA2.Total, PBRM1.Truncal and SMARCA4.Truncal all showed statistically significant association with OS: the losses of ARID1A, SETD2, and PBRM1 were associated with worse prognosis for the patients, while the losses of SMARCA4 and SMARCA2 were associated with better prognosis (Table 4). Interestingly, for PBRM1 and SMARCA4, if the ITH analysis were not performed, their associations with the OS would not have been discovered. Thus ITH analysis also unearthed hidden associations between marker losses and overall survival.

We also used the CART methodology to perform multivariable analyses. The results were consistent with PH model results (Tables 3 and 4). Again, ITH analysis derived truncal losses were key to derive meaningful associations between marker losses and patient survivals in this type of association analysis.

ARID1A is a specificity subunit of the SWI/SNF chromatin-remodeling complex. Decreased ARID1A expression was prevalent, and it was statistically associated with shorter patient survivals [26]. Even though a very low percentage of ccRCC tumors harbor mutations in ARID1A, the high rate of ARID1A expression loss clearly indicates that it plays a critical role in cancer biology in ccRCC, and a clever way to take advantage of its loss to treat ccRCC is worth serious efforts to pursue. Decreased expression of SETD2 was also linked to unfavorable prognosis for patients with nonmetastatic ccRCC [27]. Both were consistent with our result. PBRM1 is another specificity subunit of the SWI/SNF complex. The contribution of PBRM1 mutations to the clinical outcome of ccRCC patients has been somewhat controversial [28–31]. Our analysis strongly suggests that PBRM1 loss is enriched at higher tumor stages (Figure 2) and is strongly associated with worse overall survival (Table 4 and Figure 4). SMARCA4 and SMARCA2 are two mutually exclusive catalytic subunits of the SWI/SNF complex. Their protein losses in ccRCC were unknown. We found that SMARCA4 and SMARCA2 had prevalent expression loss in ccRCC tumors (Table 2). In both multivariable analyses, the SMARCA2 loss was strongly associated with longer patient overall survival. In Small Cell Carcinoma of the Ovary, Hypercalcaemic Type (SCCOHT) and Non-Small Cell Lung Cancer cell cells, SMARCA4 and SMARCA2 acted as tumor suppressors [19, 32]. Thus the tumor-promoting functions of SMARCA4 and SMARCA2 might be unique to ccRCC. Although the oncogenic pathways activated by SMARCA4 and SMARCA2 in the absence of PBRM1 or ARID1A are currently unknown, they can and should be identified in ccRCC cells and tumors with PBRM1 or ARID1A deficiency. The identification of these oncogenic pathways will prove useful to rationally design therapeutic strategies to treat ccRCC tumors with PBRM1 or ARID1A deficiency.

## MATERIALS AND METHODS

### Sample preparation and TMA preparation

A protocol approved by Fox Chase Cancer Center IACUC committee (IRB#13-810) was used to obtain written informed patient consent. Institutional guidelines and protocols were strictly followed when all samples were collected.

160 Patients diagnosed with clear cell renal cell carcinoma with available archived Paraffin fixed tissue were identified from Fox Chase cancer Center kidney database. 40 cases from each of the four tumor stages (Stage I–IV) were randomly picked. A pathologist reviewed all cases. From each tumor, four different areas were selected to cover the intratumoral heterogeneity. Eight tissue microarray blocks (TMA) were built at Fox Chase Cancer Center biorepository facility.

### Immunohistochemistry and scoring

The Ventana Discovery ULTRA staining platform with Discovery CCI (Ventana cat#950-500) was used for antigen retrieval. The total application time was 64 minutes. Primary immunostaining step utilized antibodies against PBRM1 1:50, ARID1A 1:250, SMARCA2 1:50, SMARCA4 1:200, SEDT2 1:100 in Ventana Antibody Dilution Buffer (Ventana cat #ADB250). The slides were incubated for 44 minutes at room temperature. Secondary immunostaining was done with a rabbit Horseradish Peroxidase (HRP) multimer cocktail (Ventana cat#760-500). The immune complexes were developed with the ultraView Universal DAB (diaminobenzidine tetrahydrochloride) Detection Kit (Ventana cat#760-500). After this the slides were washed with a Tris based reaction buffer (Ventana cat#950-300) and stained for 8 minutes with Hematoxylin II (Ventana cat #790-2208). The antibodies used for IHC are: PBRM1 (Bethyl labs, Cat# A301-591A), ARID1A (Sigma-Aldrich, Cat# HPA005456), SMARCA2 (Sigma-Aldrich, Cat# HPA029981), SMARCA4 (Abcam, Cat# ab110641), SETD2 (ProSci, Cat# 30-305).

Two pathologists (W.J., T.P.) performed the scoring of the stained foci independently. A score of 2 is given if greater than 50% of tumor cells were considered positive in a focus, 1 if less than 50% but greater than 5% of tumor cells were deemed positive, and 0 if less than 5% of tumor cells were positive. In the cases where the two pathologists gave different scores, they examined the foci together to reach a consensus. If one marker is scored as 0 in one focus, then that whole tumor is deemed to have a score of 0 for that marker.

### Statistical analysis

Categorical variables were compared between groups using Fisher's exact tests. Univariate and multivariable Cox proportional hazards (PH) models were used to associate overall survival (OS) and recurrence-free survival (RFS) with grade, stage and biomarkers of interest. The markers included Arid1A.Total, SetD2.Total, SMARCA4.Total, SMARCA2.Total, PBRM1.Truncal, SetD2.Truncal, SMARCA4.Truncal and SMARCA2.Truncal. Estimates of hazard ratio (HR) including 95% confidence intervals were computed for each variable. Goodness-of-fit of the Cox PH model was assessed using Schoenfeld residuals [33]. For variables showing a time-varying effect on survival, weighted Cox regression methods were used to account for these effects by computing average HRs [34]. In addition, multivariable analyses were performed by constructing decision trees using the classification and regression trees (CART) methodology. A decision tree is a logical model represented as a binary tree that shows how the value of a response variable such as OS or RFS can be predicted by using the values of a set of clinical variables and biomarkers. The unified CART framework that embeds recursive binary partitioning into the theory of permutation tests was used [35]. This approach overcomes the problem of over-fitting and selection bias towards variables with many possible splits or missing values. It utilizes significance testing procedures and results in unbiased selection among variables measured at different scales. All tests were two-sided and used a Type I Error of 5% to determine statistical significance. Computations were performed in the R statistical language and environment using packages *survival* and *party* [36].

## Abbreviations

(ITH): Intratumoral heterogeneity
(IHC): Immuno histochemistry
(TMA): Tissue microarray
(ccRCC): Clear cell Renal Cell Carcinoma
(PTEN): Phosphatase And Tensin Homolog
(KDM5C/JARID1C): Lysine Demethylase 5C
(PBRM1): Polybromo-1
(BAP1): BRCA1-associated protein 1
(SETD2): SET domain containing 2
(ARID1A): AT-Rich Interaction Domain 1A
(SMARCA4/BRG1): SWI/SNF Related, Matrix Associated, Actin Dependent Regulator Of Chromatin, Subfamily A, Member 4
(SMARCA2/BRM): SWI/SNF Related, Matrix Associated, Actin Dependent Regulator Of Chromatin, Subfamily A, Member 2
(CART): Classification and regression trees
(VHL): von-Hippel Lindau tumor suppressor
(NGS): Next Gen Sequencing
(RFS): Recurrence-free survival
(OS): Overall survival
(CML): Chronic myeloid leukemia
(GIST): Gastrointestinal stromal tumors
(SCCOHT): Small Cell Carcinoma of the Ovary, Hypercalcaemic Type
